# Targeting microRNA methylation: Innovative approaches to diagnosing and treating hepatocellular carcinoma

**DOI:** 10.1016/j.ncrna.2024.12.002

**Published:** 2024-12-14

**Authors:** Albert Sufianov, Murad Agaverdiev, Andrey Mashkin, Tatiana Ilyasova

**Affiliations:** a Educational and Scientific Institute of Neurosurgery, Рeoples’ Friendship University of Russia (RUDN University), Moscow, Russia; b Department of Neurosurgery, Sechenov First Moscow State Medical University (Sechenov University), Moscow, Russia; c Bashkir State Medical University, Ufa, Republic of Bashkortostan, 3 Lenin Street, 450008, Russia

**Keywords:** Hepatocellular carcinoma, MicroRNA, Methylation, Diagnostic biomarkers, Therapeutic targets, Cancer therapy

## Abstract

Hepatocellular carcinoma (HCC) stands as the most prevalent form of primary liver cancer and is frequently linked to underlying chronic liver conditions such as hepatitis B, hepatitis C, and cirrhosis. Despite the progress achieved in the field of oncology, HCC remains a significant clinical challenge, primarily due to its typically late-stage diagnosis and the complex and multifaceted nature of its tumor biology. These factors contribute to the limited effectiveness of current treatment modalities and result in poor patient prognosis. Emerging research has underscored the vital role of microRNAs (miRNAs)—small, non-coding RNA molecules that play a pivotal part in the post-transcriptional regulation of gene expression. These miRNAs are integral to a wide array of cellular functions, including proliferation, apoptosis, and differentiation, and their dysregulation is closely associated with the pathogenesis of various cancers, notably HCC. A major focus in recent studies has been on the epigenetic regulation of miRNAs through methylation, a key mechanism that modulates gene expression. This process, involving the addition of methyl groups to CpG islands in the promoter regions of miRNA genes, can result in either gene silencing or activation, influencing the expression of oncogenes and tumor suppressor genes. Such alterations have profound implications for tumor growth, metastasis, and resistance to treatment. Evidence suggests that aberrant miRNA methylation can serve as a powerful biomarker for early detection and prognosis in HCC and may present novel opportunities for therapeutic intervention. This review aims to provide a comprehensive overview of the current landscape of miRNA methylation in HCC, elucidating its significance in the molecular mechanisms of liver cancer and examining its potential for clinical application. By exploring the diagnostic and therapeutic potential of miRNA methylation, we seek to highlight its value in enhancing personalized treatment strategies and improving patient outcomes.

## 1 Introduction

**Fig. 1:**
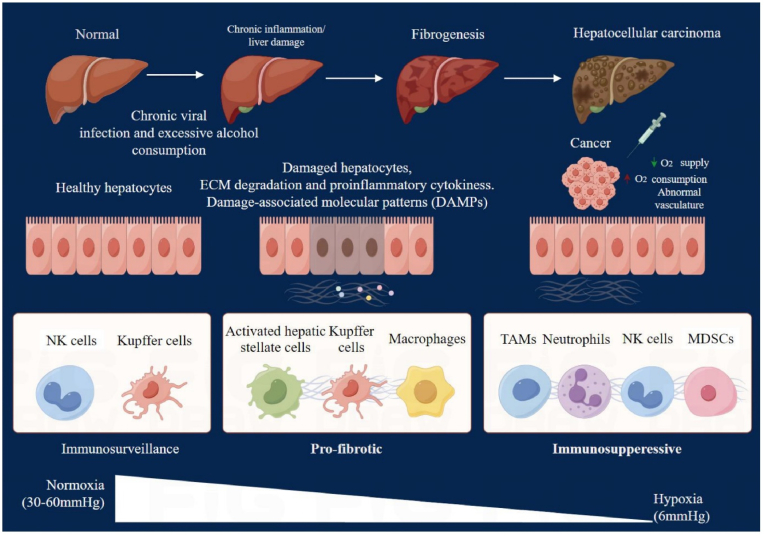
Development of hepatocellular carcinoma (HCC). This figure illustrates the progressive stages of HCC development, detailing the role of chronic liver diseases, viral infections (notably hepatitis B and C), and cirrhosis in promoting cellular changes that lead to malignancy. It highlights molecular and cellular events such as DNA damage, oxidative stress, and mutations that trigger the carcinogenic process. The figure also emphasizes the involvement of specific signaling pathways and genetic predispositions that accelerate the progression to full-blown HCC, providing a comprehensive visualization of the disease's etiology.

Hepatocellular carcinoma (HCC) is the sixth most prevalent cancer globally and the third leading cause of cancer-related mortality, accounting for approximately 750,000 deaths annually [1]. The burden of this disease is especially high in China, where factors such as hepatitis B prevalence, liver cirrhosis, aflatoxin exposure, and chemical toxins contribute to nearly half (47 %) of the global incidence [2]. The prognosis for HCC remains grim, largely because the majority of patients present at an advanced stage of the disease, complicating treatment efforts and diminishing survival outcomes (Fig. 1).

In response to these challenges, there is an urgent need to explore new diagnostic and therapeutic strategies that can improve patient prognosis and manage the disease more effectively. Over the last decade, epigenetic modifications have emerged as significant factors in the oncogenesis and progression of HCC. Among these, promoter methylation, particularly in the context of microRNAs (miRNAs), plays a critical role. The aberrant methylation of CpG islands within miRNA genes can alter miRNA expression, which in turn impacts the expression and functionality of target oncogenes or tumor suppressor genes, thereby influencing the overall biological behavior of HCC tumors. This review article delves into the pivotal findings from recent research on miRNA methylation, highlighting its implications in the occurrence, diagnosis, prognosis, and clinical treatment of HCC. By examining these developments, we aim to provide new insights and potential approaches for enhancing the clinical management of this formidable cancer.

## 2 Overview of miRNA methylation

miRNAs are a category of intrinsic, non-coding, single-stranded RNAs about 22 nucleotides in length that are critical in regulating gene expression by targeting specific messenger RNAs (mRNAs). This targeting can lead to the degradation of mRNAs or suppression of their translation process [3]. The maturation of miRNAs begins with the synthesis of primary miRNA (pri-miRNA), which is subsequently processed into precursor miRNA (pre-miRNA) by the Drosha/DGCR8 heterodimer in the nucleus. This precursor is then transported to the cytoplasm via a complex with Exportin 5 and its catalytic partner Ran-GTP. In the cytoplasm, the RNase III enzyme Dicer cleaves the pre-miRNA into a roughly 22-nucleotide long mature miRNA duplex, which is then unwound by helicases to produce functional single-stranded miRNA [4].

**Fig. 2:**
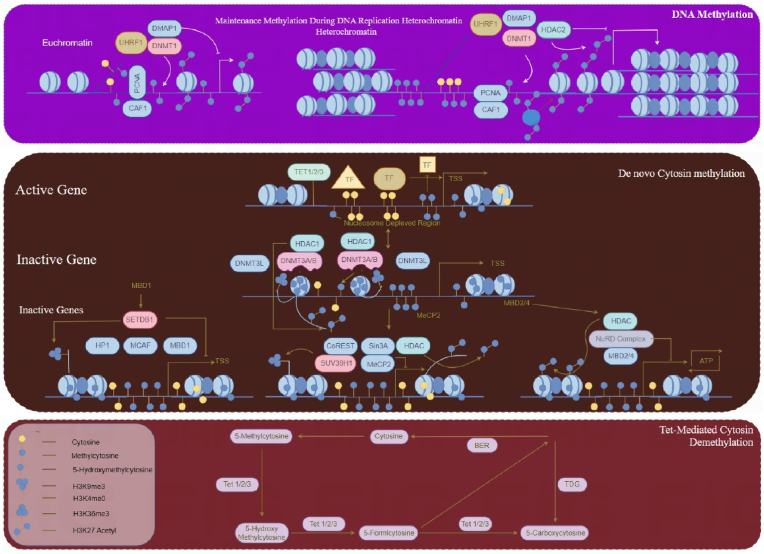
DNA methylation mechanism. The figure represents the biochemical process of DNA methylation, focusing on how methyl groups are added to CpG islands in the promoter regions of genes, impacting transcription. It showcases how hypermethylation results in the silencing of tumor suppressor genes, while hypomethylation may activate oncogenes, thereby contributing to carcinogenesis. This visual aid underlines the role of DNA methyltransferases (DNMTs) and other regulatory proteins in the epigenetic landscape, crucial for understanding miRNA regulation and its implications in hepatocellular carcinoma (HCC).

Increasing evidence has linked the aberrant expression of miRNAs to a wide array of diseases, including various cancers, inflammatory conditions, and autoimmune disorders. These miRNAs have potential utility as biomarkers for early detection, prognosis, and therapeutic intervention. In recent studies, it has been observed that the expression of miRNAs is governed by epigenetic mechanisms such as promoter methylation and histone modification, which are intimately connected to disease pathogenesis and progression [5]. Approximately half of miRNA genes are associated with CpG islands, highlighting the significant role of DNA methylation in controlling miRNA expression [6]. Aberrant DNA methylation can be categorized into hypomethylation, which leads to increased gene expression due to the loss of methylation marks, and hypermethylation, which involves the gain of methylation marks at previously unmethylated sites, typically resulting in stable transcriptional repression and reduced gene expression [7] (Fig. 2).

As research into tumors deepens, it has become apparent that miRNA genes frequently reside within cancer-associated genomic regions and can play dual roles as oncogenes or tumor suppressor genes [8]. The regulation of miRNA by abnormal DNA methylation involves both the activation of oncogenes and the silencing of tumor suppressor genes through DNA hypomethylation and hypermethylation, respectively. This intricate interplay further elucidates the mechanisms of tumor cell carcinogenesis and offers potential targets for therapeutic intervention [9]. This comprehensive understanding of miRNA regulation and its implications in disease states emphasizes the necessity for ongoing research in this dynamic field, potentially paving the way for novel diagnostic and therapeutic strategies.

## 3 miRNA methylation and the progression and development of HCC

**Fig. 3:**
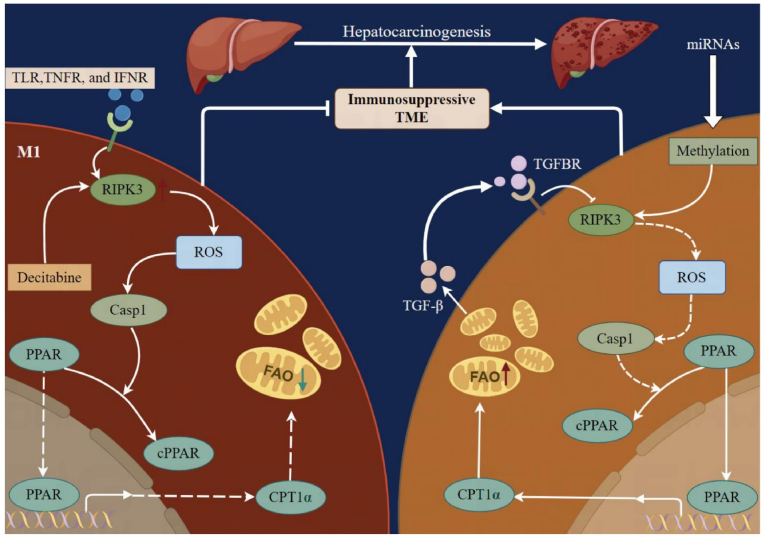
Pathogenesis and Role of microRNAs (miRNAs) in hepatocellular carcinoma (HCC). This detailed figure maps the contribution of microRNAs to the pathogenesis of HCC, emphasizing their dual roles as tumor suppressors and oncogenes. It illustrates how viral proteins, such as HBx from hepatitis B virus, induce abnormal miRNA methylation, leading to either silencing or overexpression. The figure also demonstrates miRNA interactions with signaling pathways like PI3K/Akt and NOTCH, highlighting their regulation of tumor growth, angiogenesis, and metastasis. It underscores how epigenetic changes in miRNAs contribute to altered gene expression, influencing the progression and treatment resistance of HCC.

In recent studies, miRNA methylation has been increasingly recognized as a key factor in the development of HCC (Fig. 3).

The hepatitis B virus (HBV) and various biochemical toxins have been implicated in driving HCC progression through the aberrant methylation of specific miRNAs. It has been estimated that HBV infection is responsible for 70 %–80 % of HCC cases, with the hepatitis B virus X protein (HBx) playing a crucial role in this process. HBx influences the regulation of several epigenetic mechanisms, including miRNA methylation and acetylation, which disrupt multiple cellular pathways and functions [10]. MiR-132, which is produced from an intergenic region on chromosome 17 via the transcription factor cAMP response element-binding protein, has been identified as being underexpressed in HCC [11]. Research has demonstrated that miR-132 acts as a tumor suppressor in HCC by deactivating the Akt signaling pathway, particularly through the reduction of Akt phosphorylation and cyclin D1 levels. This suppression is inversely associated with HBx expression in HCC tissues. It is suggested that HBx interacts with DNA methyltransferase (DNMT), leading to the hypermethylation of the miR-132 promoter and the subsequent silencing of its expression. This epigenetic alteration is critical in fostering the onset and rapid progression of HCC [12]. Similarly, miR-205, which has been found to be downregulated in various cancers [[13], [14], [15]], targets HBx directly and inhibits its expression, thus exerting a tumor-suppressive effect in HCC tissues. Further investigations have revealed that HBx may counteract this by inducing hypermethylation of the miR-205 promoter, effectively neutralizing its tumor-suppressive activity and promoting the proliferation of HCC cells [16]. Moreover, miR-137 has been shown to reduce the proliferation of MHCC97H cells treated with HBx-siRNA by targeting Notch1 [17]. Notch1 is known to be involved in promoting angiogenesis and the epithelial-mesenchymal transition in HCC cells, through modulation of E-cadherin, which further facilitates the invasion and recurrence of HCC [18,19]. HBx has been found to suppress miR-137 expression by inducing methylation of the miR-137 gene, thereby enhancing the pathogenicity and progression of HCC [17]. Overall, the epigenetic repression of miRNA expression induced by HBx-mediated DNA methylation provides crucial insights into the mechanisms by which HBV contributes to hepatocarcinogenesis, underscoring the importance of understanding and targeting these epigenetic changes in the fight against HCC.

miRNA methylation has emerged as a crucial factor in the development of HCC linked to exposure to biochemical toxins. Mycotoxins, commonly found in crops, are recognized as significant carcinogens and pose a major risk for liver cancer, particularly in sub-Saharan Africa [20]. Fusaric acid (FA), a mycotoxin present in moldy corn, is known for its toxicity to both animals and plants [21]. Research into the role of epigenetic modifications in the toxicity of FA has revealed that FA can induce global DNA hypomethylation in HepG2 liver cancer cells. This occurs through the downregulation of DNA methyltransferases DNMT1, DNMT3A, and DNMT3B, and the upregulation of Methyl CpG Binding Domain Protein 2 (MBD2), a marker often associated with cancer due to its role in causing genomic instability and increasing mutation frequencies [22]. Further investigations indicate that FA promotes the upregulation of miR-29b in HepG2 cells by demethylating its promoter region. miR-29b can then target DNMT3A and DNMT3B directly, and DNMT1 indirectly by suppressing the transcriptional activator Sp1, leading to reduced expression of these DNMTs and consequently, global DNA hypomethylation. This elucidates an epigenetic regulatory mechanism for FA-induced genotoxicity and cytotoxicity [23].

Moreover, other organic chemical contaminants like Trichlorethylene (TCE), a widely used volatile organic solvent, have been implicated in liver cancer. It has been noted that exposure to TCE can cause upregulation of the oncogenic miRNA, miR-182-5p, in liver tissues by reducing the methylation capabilities of DNMT3A and DNMT3B [[24], [25], [26]]. The increased expression of miR-182-5p inhibits Cited2, a tumor suppressor gene, thereby enhancing cellular proliferation and contributing to the progression of TCE-related HCC [27]. Another chemical, Furan, found commonly in heated foods, has been classified as a potential human carcinogen [28]. Studies have shown that miR-375, a miRNA with tumor-suppressing properties, is consistently downregulated in liver cancer induced by furan. This downregulation is linked to hypermethylation of the histones H3K9 and H3K27 that bind to miR-375, further implicating miRNA methylation in the pathogenesis of liver cancer (Table 1) [29,30].

**Table 1:** MiRNA methylation and its impact on HCC.

miRNA	Mechanism	Function	Impact on HCC	Reference
**miR-132**	HBx induces hypermethylation of miR-132 promoter, silencing its expression.	Acts as a tumor suppressor by deactivating Akt signaling pathway, inversely associated with HBx expression.	Promotes HCC onset and progression.	[11]
**miR-205**	HBx induces hypermethylation of miR-205 promoter, neutralizing its tumor-suppressive activity.	Targets HBx directly, inhibiting its expression and exerting tumor-suppressive effects.	Promotes HCC cell proliferation.	[[13], [14], [15], [16]]
**miR-137**	HBx induces methylation of miR-137 gene, suppressing its expression.	Reduces proliferation of MHCC97H cells by targeting Notch1, suppresses angiogenesis and epithelial-mesenchymal transition.	Enhances pathogenicity and progression of HCC.	[17]
**miR-29b**	FA induces demethylation of miR-29b promoter, promoting its upregulation.	Upregulated by FA, targets DNMT3A and DNMT3B, leading to global DNA hypomethylation.	Contributes to genotoxicity and cytotoxicity in HepG2 cells.	[23]
**miR-182-5p**	TCE reduces methylation capabilities of DNMT3A and DNMT3B, upregulating miR-182-5p.	Upregulated by TCE exposure, inhibits tumor suppressor Cited2, enhancing cellular proliferation.	Enhances progression of TCE-related HCC.	[24,25,27]
**miR-375**	Furan causes hypermethylation of histones binding to miR-375, leading to its downregulation.	Tumor-suppressing properties, downregulated in liver cancer induced by furan through hypermethylation of histones H3K9 and H3K27.	Contributes to liver cancer pathogenesis induced by furan.	[[28], [29], [30]]

## 4 miRNA methylation and the clinical diagnosis and prognosis of HCC

Currently, alpha-fetoprotein (AFP) remains the most utilized serum biomarker in liver cancer, playing a critical role in its screening, diagnosis, and prognostic assessment. However, AFP has shown limited sensitivity in the clinical detection of early-stage liver cancer, and its levels can also be elevated in conditions such as chronic hepatitis and cirrhosis, which has led to some debate regarding its effectiveness in clinical settings [[31], [32], [33]]. Fortunately, recent research has suggested that miRNA methylation biomarkers could potentially enhance the accuracy of diagnosing and predicting the outcome of liver cancer. These studies indicate that miRNA methylation profiles may offer a more precise and reliable means of identifying liver cancer at various stages, providing a promising alternative to traditional biomarkers, and potentially improving patient management and treatment outcomes.

### 4.1 Relationship between miRNA hypermethylation and HCC diagnosis

A considerable volume of research has established those numerous miRNAs function as tumor suppressor genes throughout the progression of HCC, and that the epigenetic silencing of these miRNAs through hypermethylation is a fundamental mechanism in the onset and progression of HCC, influencing tumor growth, metastasis, and angiogenesis [34]. The miR-200 family, consisting of five members located on chromosomes 1p36 (miR-200a, miR-200b, and miR-429) and 12p13.3 (miR-200c and miR-141), plays a crucial role in regulating the epithelial-mesenchymal transition, a key process in tumor invasion and dissemination, and is a significant step in the metastasis of primary tumors to distant sites [35,36]. DNA methylation of CpG islands is one of the key mechanisms leading to the dysregulation of the miR-200 family. It has been noted that miR-200b is partially silenced through DNA methylation, which allows it to inhibit tumor growth and invasion by directly targeting BMI1 and ZEB1 in HCC [37,38]. Moreover, the downregulation of miR-200b expression, linked to hypermethylation, has been significantly correlated with frequent recurrence (HR = 2.49) and higher mortality (HR = 2.76) in HCC patients, highlighting its unique role in initiating and maintaining the cancer stem cell population [38]. Additionally, the long non-coding RNA GIHCG, which is highly expressed in HCC, is associated with poorer survival outcomes. It has been discovered that GIHCG acts as an oncogene by increasing the trimethylation and DNA methylation levels of the miR-200b/a/429 promoter H3K27, ultimately suppressing the expression of these miRNAs to promote the proliferation and metastasis of HCC cells [39]. Beyond the miR-200 family, several other miRNAs have been identified as playing similar roles in the etiology and progression of HCC. For instance, high methylation of the MiR-192 promoter leads to the silencing of miR-192-5p, which helps maintain HCC stem cell characteristics and activates a subpopulation of hepatocytes capable of tumor initiation, thus facilitating HCC progression and contributing to poor prognosis in patients [40]. MiR-142 is found to be hypermethylated in HCC, and the methylation inhibitor 5-azacytidine (5-Aza) can restore miR-142 expression to inhibit proliferation, epithelial-mesenchymal transition, and pro-angiogenesis in a TGF-β-dependent manner in HCC, which also partially explains why reduced expression of miR-142 is linked to poor clinical outcomes in HCC [41]. Additionally, the downregulation of MiR-639 in HCC cells and tissues is partly due to hypermethylation of the miR-639 promoter by DNMT3A, with the silencing of miR-639 reducing its inhibitory effect on MYST2 and ZEB1, thereby enhancing the proliferation, migration, and invasion of HCC cells [42]. Moreover, miRNAs such as miR-9, miR-122, miR-124, miR-129, and miR-146 are downregulated through DNA hypermethylation, acting as tumor suppressors in HCC [[43], [44], [45], [46], [47]]. The relationship between miRNA methylation and the onset, progression, and prognosis of liver cancer is well-established, although most studies have been conducted at the cellular level. Larger clinical datasets are still required to validate the potential of DNA methylation as a biomarker for early diagnosis and prognosis assessment in HCC patients.

### 4.2 Relationship between miRNA hypermethylation and HCC prognosis

Additionally, various miRNAs induced by DNA hypomethylation have been observed in HCC tissues and cells, where they function as oncogenes to advance the development and occurrence of HCC. These miRNAs also serve as diagnostic and prognostic biomarkers and present potential therapeutic targets for liver cancer treatment, underscoring their significant role in medical research. Reports have highlighted that the chromosome 19 miRNA cluster (C19MC) encompasses 59 mature miRNAs, identified as critical elements in HCC pathogenesis [48,49]. Kaplan-Meier survival analysis of these miRNAs revealed a negative correlation between the expression levels of miR-512-1, miR-516a-1, and miR-519a-2 and overall survival (OS) in HCC patients. Further analyses using COX proportional hazards models indicated that high expression levels of miR-512-1 and miR-516a-1 are independent risk factors for reduced OS in HCC patients, with hazard ratios (HRs) of 1.521 and 1.662, respectively. Additionally, ROC curve analysis demonstrated that incorporating these three miRNAs into a prognostic model alongside T stage improved the AUC from 0.728 to 0.76. The promoter regions of these miRNAs exhibited consistent hypomethylation, suggesting that their upregulated expression related to HCC prognosis is driven by hypomethylation [50]. Moreover, DNA hypomethylation leading to elevated levels of hsa-miR-21-5p has been associated with poorer OS in patients with six different types of tumors, including HCC, where the HR for HCC patients are 1.6, highlighting the significant prognostic impact of miR-21 hypomethylation in HCC [51]. The role of miR-429 in HCC, however, remains contentious. Studies have shown that miR-429 can enhance HCC cell migration and invasion by directly targeting the PTEN/PI3K/AKT/β-catenin pathway, with insufficient methylation in its promoter region believed to be a key initiator of these processes [52]. This contrasts with previous findings that suggest miR-429 expression is downregulated in HCC tissues and cells, leading to speculation that miR-429 might exhibit varying effects at different stages of HCC development [39]. In conclusion, the roles played by different miRNAs in cancer progression can vary significantly, with the same miRNA potentially having divergent effects in different cancers or stages of cancer development. Abnormal miRNA methylation stands out as a crucial indicator impacting the development and progression of liver cancer, playing a key role in early screening, diagnosis, and prognosis of the disease. Despite their promise, miRNA methylation biomarkers are currently limited to laboratory settings, and their specificity and sensitivity require further validation. Consequently, there is a pressing need to identify more reliable methylation biomarkers that can effectively signal the early onset and progression of liver cancer.

## 5 miRNA methylation drugs and HCC therapy

Current clinical guidelines advise surgical resection for HCC patients who have no distant metastases and maintain robust liver function. When HCC is unresectable, treatment strategies typically include local ablation, hepatic artery intervention, targeted therapy, systemic therapy, and liver transplantation. In this context, promoter DNA hypermethylation emerges as an important biomarker and potential target for demethylating agents in the treatment of HCC [53]. The significant role of miRNAs in liver cancer has led to the exploration of miRNA methylation biomarkers as potential therapeutic targets. Given the limited number of clinical studies addressing miRNA methylation modifications for treating HCC, the discussion here focuses on three innovative miRNA methylation-related treatments for HCC. Dendritic nanocurcumin (DNC) is a formulation that enhances the therapeutic properties of curcumin—a biphenyl compound derived from turmeric rhizomes known for its anti-inflammatory, antioxidant, and antitumor effects [54]. Despite curcumin's potential in inducing DNA methylation changes, its low bioavailability has restricted its clinical use. However, dendrosomal nanocurcumin improves the absorption of curcumin into cancer cells without causing adverse effects and has shown promise in delaying HCC progression by adjusting disrupted epigenetic mechanisms, thereby establishing a basis for further clinical applications [55,56]. In laboratory settings, DNC has been shown to reactivate the expression of miR-34s by inhibiting DNMT1, DNMT3A, and DNMT3B in HepG2 and Huh7 cell lines, reducing the viability of these cancer cells [56]. Moreover, DNC-induced overexpression of miR-29a and miR-185 has been observed to suppress DNMT1, DNMT3A, and DNMT3B expression, which in turn enhances the expression of the long non-coding RNA MEG3 in HCC cells through DNA methylation mechanisms [57]. These findings indicate that DNC can promote the overexpression of certain miRNAs to hinder HCC progression. Although these studies highlight changes in DNMT expression, they do not directly address the methylation status of miRNAs. More comprehensive research is necessary to establish a clear connection between DNC, miRNA methylation, and its clinical implications, especially in the context of HCC treatment. Castimycin (CAS) is a polymethylflavonoid derived from grapefruit, noted for its array of pharmacological effects, including anti-cancer properties. Studies have identified miR-148a-3p as a tumor-suppressive miRNA that is both expressed and silenced across various tumors, including HCC [[58], [59], [60]]. The interaction between miR-148a-3p and DNA methyltransferase 1 (DNMT1) plays a critical role in regulating and maintaining the stem-like properties of HCC cells. Castimycin has been shown to inhibit the activity of DNMT1, leading to the demethylation and increased expression of miR-148a-3p, which in turn suppresses the stemness traits of HCC cells [60]. Thus, Castimycin holds potential as an adjunctive treatment for liver cancer, though more comprehensive clinical studies are needed to confirm its efficacy. Arsenic Trioxide (ATO) is a traditional remedy that has been used effectively against various cancers, particularly acute promyelocytic leukemia. It is known to induce mitochondria-mediated apoptosis in cancer cells [[61], [62], [63]]. Recent studies exploring the epigenetic effects of ATO in HCC have discovered that it activates miR-148a through DNA demethylation, which subsequently diminishes the expression of NF-κB by targeting p65. This action helps to curb the cancer stem cell (CSC) phenotype, increasing the sensitivity of HCC cells to chemotherapeutic agents like 5-fluorouracil (5-FU) and oxaliplatin [64]. This mechanism offers a promising new avenue for enhancing the efficacy of existing HCC treatments. Moreover, it has been demonstrated that the methylation-regulated miR-193a-3p enhances HCC resistance to 5-FU by targeting and suppressing the expression of SRSF2, a splicing factor [65]. Collectively, these findings underline the potential of miRNA methylation drugs to combat liver cancer by reducing genomic or specific miRNA methylation levels. Targeting DNMT activity and altering DNA methylation patterns has emerged as a novel strategy in the utilization of miRNAs for HCC treatment, highlighting a growing area of research with significant therapeutic implications (Table 2).

**Table 2:** MiRNA methylation in HCC diagnosis and therapy.

Aspect	Function/Role	Mechanism	Impact	Reference
**Current Biomarker**	AFP used for screening, diagnosis, and prognosis of liver cancer.	Limited sensitivity for early-stage liver cancer, elevated in chronic hepatitis and cirrhosis.	Debate on effectiveness due to sensitivity and specificity issues.	[31]
**miR-200 Family**	Regulates epithelial-mesenchymal transition; miR-200b inhibits tumor growth and invasion.	DNA methylation of CpG islands silences miR-200b, linked to recurrence and mortality.	Silencing promotes tumor proliferation and metastasis.	[35]
**miR-192**	Silencing maintains HCC stem cell characteristics, contributing to poor prognosis.	High promoter methylation silences miR-192-5p.	Facilitates HCC progression, poor prognosis.	[40]
**miR-142**	Restored by 5-Aza, inhibits proliferation and epithelial-mesenchymal transition.	Hypermethylated in HCC, 5-Aza restores expression.	Linked to poor clinical outcomes.	[41]
**miR-639**	Downregulation enhances proliferation, migration, and invasion of HCC cells.	Hypermethylated by DNMT3A, silencing reduces inhibitory effect on MYST2 and ZEB1.	Promotes HCC cell proliferation, migration, and invasion.	[42]
**C19MC Cluster**	Associated with reduced OS in HCC patients; driven by hypomethylation.	Promoter hypomethylation leads to upregulation.	Independent risk factors for reduced OS in HCC.	[48]
**hsa-miR-21-5p**	Hypomethylation associated with poorer OS in various tumors including HCC.	Upregulated due to hypomethylation.	Significant prognostic impact in HCC.	[51]
**miR-429**	Enhances cell migration and invasion, conflicting findings on its expression.	Insufficient methylation initiates PTEN/PI3K/AKT/β-catenin pathway.	Varying effects at different stages of HCC development.	[52]
**Dendritic Nanocurcumin (DNC)**	Improves curcumin absorption, reactivates miR-34s, overexpresses miR-29a and miR-185.	Inhibits DNMT1, DNMT3A, and DNMT3B, enhancing miRNA expression.	Promotes miRNA expression to hinder HCC progression.	[54]
**Castimycin (CAS)**	Inhibits DNMT1, demethylates miR-148a-3p, suppresses HCC stemness traits.	Inhibits DNMT1 activity, increases miR-148a-3p expression.	Suppresses stemness traits of HCC cells.	[[58], [59], [60]]
**Arsenic Trioxide (ATO)**	Activates miR-148a, diminishes NF-κB expression, increases chemotherapy sensitivity.	Induces miR-148a demethylation, reduces CSC phenotype.	Enhances efficacy of HCC treatments.	[[61], [62], [63]]

## 6 Conclusion

The past decade has witnessed remarkable advancements in the understanding of miRNA methylation and its role in HCC, offering a new dimension to the epigenetic regulation of cancer. The dysregulation of miRNA expression through hypermethylation or hypomethylation has been identified as a critical factor influencing the initiation, progression, and metastasis of HCC. These epigenetic modifications can either promote oncogenesis by silencing tumor suppressor miRNAs or facilitate tumor suppression by downregulating oncogenic miRNAs. The complex interplay between miRNA methylation and gene expression has significant implications for the biological behavior of HCC, including its growth patterns, invasiveness, and response to therapies. Recent studies have shown promise in the development of targeted therapies that modulate miRNA methylation. Approaches such as dendritic nanocurcumin and castimycin have been demonstrated to inhibit DNA methyltransferase activity, leading to the reactivation of silenced tumor suppressor miRNAs and the restoration of normal cellular functions. These epigenetic therapies have the potential to reshape the tumor microenvironment, enhance the efficacy of existing treatments, and reduce the risk of disease recurrence. Additionally, the use of arsenic trioxide to demethylate specific miRNAs has shown potential in increasing the sensitivity of HCC cells to chemotherapy, suggesting a viable strategy for overcoming drug resistance. However, despite these promising developments, the translation of miRNA methylation research into clinical practice is still in its early stages. The current limitations include a lack of large-scale clinical trials, challenges in ensuring the specificity and safety of epigenetic therapies, and the need for more precise methods of miRNA methylation analysis. To advance the clinical utility of miRNA methylation, future research must focus on integrating genomic, epigenetic, and transcriptomic data to create a more comprehensive understanding of HCC. This integrated approach can pave the way for the development of highly personalized diagnostic and therapeutic strategies, enabling clinicians to identify and target specific epigenetic changes unique to each patient's tumor profile.

The future of HCC management lies in leveraging the potential of miRNA methylation to develop targeted therapies and refine diagnostic techniques. Advancements in next-generation sequencing and bioinformatics tools are expected to play a crucial role in enhancing the precision and scalability of miRNA methylation studies. The continued exploration of miRNA methylation as both a biomarker and a therapeutic target holds the promise of significantly improving early detection, treatment outcomes, and overall patient survival. By addressing current gaps in research and advancing the translation of epigenetic findings into clinical practice, the field can move towards more effective and personalized approaches to the treatment and management of hepatocellular carcinoma.

## CRediT authorship contribution statement

**Albert Sufianov:** Project administration, Methodology, Investigation, Conceptualization. **Murad Agaverdiev:** Writing – review & editing, Writing – original draft. **Andrey Mashkin:** Visualization, Validation, Resources. **Tatiana Ilyasova:** Validation, Software, Resources.

## Funding

This work was supported by the Bashkir State Medical University Strategic Academic Leadership Program (PRIORITY-2030).

## Declaration of competing interest

All authors declare that there are no competing interests.

## LIST OF ABBREVIATIONS

HCC: Hepatocellular carcinoma
MiRNA: MicroRNA
CpG: Cytosine-phosphate-Guanine
DNMT: DNA methyltransferase
CSC: Cancer stem cell
5-FU: 5-fluorouracil
ATO: Arsenic trioxide
OS: Overall survival
AFP: Alpha-fetoprotein
DNC: Dendritic nanocurcumin
CAS: Castimycin
NF-Κb: Nuclear factor kappa-light-chain-enhancer of activated B cells
SRSF2: Serine/arginine-rich splicing factor 2
PTEN: Phosphatase and tensin homolog
PI3K: Phosphoinositide 3-kinase
AKT: Protein kinase B
mRNA: Messenger RNA
pri-miRNA: Primary microRNA
pre-miRNA: Precursor microRNA
CHB: Chronic hepatitis B
